# Genotyped indigenous Kiwcha adults at high altitude are lighter and shorter than their low altitude counterparts

**DOI:** 10.1186/s40101-022-00280-6

**Published:** 2022-03-10

**Authors:** Esteban Ortiz-Prado, Gonzalo Mendieta, Katherine Simbaña-Rivera, Lenin Gomez-Barreno, Samanta Landazuri, Eduardo Vasconez, Manuel Calvopiña, Ginés Viscor

**Affiliations:** 1 One Health Research Group, Faculty of Medicine, Universidad de las Américas, Calle de los Colimes Avenida De los Granados, 170137 Quito, Ecuador; 2 Department of Cell Biology, Physiology and Immunology, Universitat de Barcelona (UB), Barcelona, Spain; 3 Universidad de las Américas, Quito, Ecuador

**Keywords:** Anthropometric; High altitude; Natives; Adaptation, Hypoxia, Weight, Height, BMI

## Abstract

**Background:**

Anthropometric measures have been classically used to understand the impact of environmental factors on the living conditions of individuals and populations. Most reference studies on development and growth in which anthropometric measures were used were carried out in populations that are located at sea level, but there are few studies carried out in high altitude populations.

**Objective:**

The objective of this study was to evaluate the anthropometric and body composition in autochthonous Kiwcha permanently living at low and high altitudes.

**Methodology:**

A cross-sectional study of anthropometric and body composition between genetically matched lowland Kiwcha from Limoncocha (*n* = 117), 230 m in the Amazonian basin, and high-altitude Kiwcha from Oyacachi (*n* = 95), 3800 m in Andean highlands. Student’s *t*-test was used to analyze the differences between continuous variables, and the chi-square test was performed to check the association or independence of categorical variables. Fisher’s exact test or Spearman’s test was used when the variable had evident asymmetries with histograms prior to the selection of the test.

**Results:**

This study shows that high altitude men are shorter than their counterparts who live at low altitude, with *p* = 0.019. About body muscle percentage, women at high altitudes have less body muscle percentage (− 24.8%). In comparison, men at high altitudes have significantly more muscle body mass percentage (+ 13.5%) than their lowland counterparts. Body fat percentage was lower among low altitude women (− 15.5%), and no differences were found among men.

**Conclusions:**

This is the first study to be performed in two genotyped controlled matching populations located at different altitudes to our best knowledge. The anthropometric differences vary according to sex, demonstrating that high altitude populations are, in general, lighter and shorter than their low altitude controls. Men at high altitude have more muscled bodies compared to their lowland counterparts, but their body age was older than their actual age.

**Supplementary Information:**

The online version contains supplementary material available at 10.1186/s40101-022-00280-6.

## Introduction

Body composition, including size, weight, and height, and body mass index (BMI) are shaped by genetic, environmental, and sociodemographic circumstances. These features can be subtle or marked, and depending on time, they can be temporal or perennial [1, 2]. At high altitude, variation reflects genomic traits selected by hypoxia-related stressors over many generations and resulting in variable adaptations and phenotypes across populations [3–8].

One of the factors associated with evolutionary changes has been the elevation at which a population resides [9]. High altitude exposure is associated with a reduced oxygen availability, utilization, or consumption that has several physiological and pathological implications among acclimatized and adapted humans [10].

These adaptations often vary from place to place and, more importantly, in how much time the exposure has had to push genetic, anatomical, morphological, or physiological changes [9, 11]. In this sense, long-term high altitude exposure has triggered many adaptation mechanisms and anthropometric differences that vary from region to region [9, 12–14].

Most reports on the adaptive mechanisms that humans have undergone in relation to the environmental conditions to which their ancestors were exposed have been studied in populations located at sea level. Nevertheless, high-altitude triggered changes are significant [15, 16].

Some adaptive changes described among high altitude populations rely on how much time has passed [17]. For instance, inhabitants from the Himalayas mountainous regions have adapted differently compared to Andean high altitude dwellers [18, 19]. Greater and wider chest as well as smaller bodies are some features of the Andean high altitude natives, while thinner and taller bodies have been described among Himalayan Sherpas [9, 17–19].

Some of these morphological and adaptive differences are evident at birth, while others can be observed at older ages [20, 21]. A study performed by Cossio-Bolaños et al. in Peru within populations located at 2320 m with those located at 3000 m above sea level [22]. The study reported that physical growth at high altitudes was affected by a small (1–4 cm) delay in linear growth and skeletal maturation [22]. There was also observation that the chest circumference among children at high altitude (4150 m) was 12 to 15% greater compared to American and Peruvian children that were born at sea level [22].

Most of those anthropometric and physiological differences between the populations living at high altitudes in different parts of the world are based on the wide differences in time; generations have passed from the initial colonization of these high altitude ecological niches [23, 24]. So, the genetic architecture of altitude-adapted human populations could play an important role in their anatomical and morphological development as a mean to better survive at high altitudes. As noted, evolutionary differences between various populations have been compared on some occasions; however, the comparisons are usually between distinct populations residing in different locations.

The main goal of this study was to compare some anthropometric variables and body composition parameters in two genetically homogeneous populations of Kiwcha, living at low and high altitudes from several generations.

## Methodology

### Study design

A cross-sectional analysis of the differences in anthropometric parameters and body composition was carried out in two populations of Kiwcha, natives from Ecuador.

### Setting

This study was carried out in Ecuador in two geographically different areas, the Andes and the Amazon basin. The research work began in January 2017 and concluded in August 2019.

**Fig. 1:**
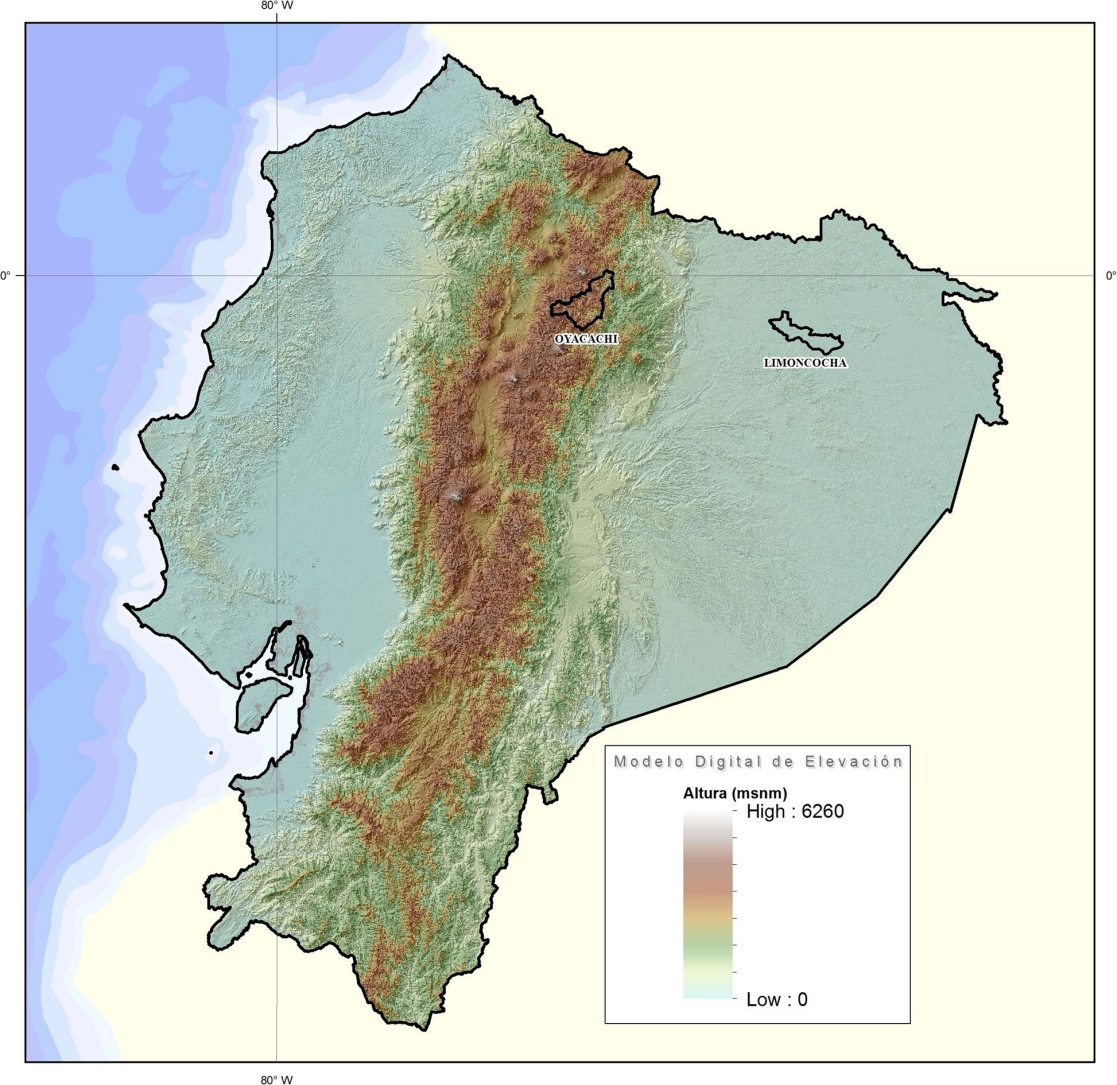
Topographic map of Ecuador highlighting Limoncocha (230 m) and Oyacachi (3800 m)

Ecuador, with an area of more than 283,000 km^2^, is the smallest country in the Andean mountainous region in South America. The country is divided into four geographical regions, the coast, the highlands, the Amazon region, and the Galápagos Islands. The political division encloses 24 provinces, ten from the highlands, seven from the coast, six from the Amazon region, and one from the insular region of Galápagos. Every province has several political divisions called cantons, and they are comparable to cities elsewhere. The country has 141 cantons at low altitude, 28 at moderate altitude, 41 at high altitude, and 11 at very high altitude. Limoncocha is located at a low altitude, while Oyacachi is located at a very high altitude (Fig. 1).

### Participants

All the participants who voluntarily agreed to participate were members of the Kiwcha indigenous group from Ecuador. The high-altitude group came from Oyacachi, a small Kiwcha community located at 3800 m of elevation, while the low-altitude group came from Limoncocha, located at 230 m of elevation.

### Inclusion criteria

The research was completed among healthy volunteers of both sexes without any type of comorbidity or chronic disease, between the ages of 18 and 85 who were born and currently residing in Oyacachi (high-altitude group) and in Limoncocha (low altitude group).

### Exclusion criteria

Volunteers who are under 18 years of age, who were born in another community and those who does not habitually reside in the parishes, were excluded from the study. Those volunteers who did not complete the anthropometric measurements were excluded from the analysis.

### Variables and outcomes

Sociodemographic variables, such as self-reported age, sex, marital status, and place of residence were recorded. We included the following anthropometric measurements: weight (kg), height (cm), body mass index (BMI), shoulder height of both arms (cm), hip height (cm), buttock height (cm), lateral arm length (cm), shoulder height in one arm (cm, median (IQR)), bi-acromial shoulder width (cm), bi-iliac width (cm), arm length (cm), chest circumference (cm), waist circumference (cm), head circumference (cm), body fat composition (%), body muscle composition (%), corporeal or body age (years), and actual age (years).

The main outcome is to determine the possible anthropometric differences between genotype-matched Kiwcha indigenous people who live at high altitudes versus their counterparts who live at low altitudes.

Age subgroup analysis between communities and gender were performed due to important variations of anthropometric measures in adults due to normal aging.

### Anthropometric measurements

All participants were measured in a private room in the presence of a chaperone but hidden from the rest of the participants to ensure their privacy. All anthropometric measurements were taken with the participants barefoot and wearing a hospital gown. We started with weight, height, and body composition measurements using the HBF-511B Body Composition Monitor from Omron® [25]. This body composition monitor, besides calculating body weight, also measures body and visceral fat as well as muscle mass percentage. Using a built-in algorithm, BMI was automatically calculated.

The different anthropometric measurements were performed in all participants who voluntarily accepted to be part of the study. The techniques used to measure the distance between anatomical references points were based on international guidelines summarized in the report by Mazza [26]. A standard measuring tape was used to obtain data from each anthropometric measure, recorded in each participant’s file and digitalized for the statistical analysis.

### Body composition

Using a built-in palm sensor from the Omron® monitor and based on the electrical bio impedance, we were able to measure the body composition of the human body, including fat and muscle percentage. Considering electric data and relating to other data such as age, sex, and height of the individual, the muscle mass and fat percentage of the whole body were obtained.

### Corporeal or body age

The calculated body age or biological age is a metric based on one’s own resting metabolism. Body age was calculated using the participant’s weight, body fat and muscle percentage and was used as a guide to determine whether the body age is above or below the actual average age of each individual [27].

### Data sources

Individual-level sociodemographic information, place of residence, and past medical history were obtained in situ in both communities. A complete physical examination including body weight, height, and anthropometric variables recording was performed.

### Study size and sample size calculation

In terms of the number of participants required to achieve significance, the sample size (*n*) and margin of error (*E*) were given by the following formula:

$$x=Z{\left(\raisebox{1ex}{$c$}\!\left/ \!\raisebox{-1ex}{$100$}\right.\right)}^2r\left(100-r\right)$$

$$n=\raisebox{1ex}{$ Nx$}\!\left/ \!\raisebox{-1ex}{$\left(\left(N-1\right){E}^2+x\right)$}\right.$$

$$\mathrm{E}=\mathrm{Sqrt}\left[\raisebox{1ex}{$\left(N-n\right)x$}\!\left/ \!\raisebox{-1ex}{$n\left(N-1\right)$}\right.\right]$$

where *N* equals the population size (*n* = 570 in Oyacachi and *n* = 890 in Limoncocha), *r* equals the fraction of expected responses (50%), and *Z*(*c*/100) equals the critical value for the confidence level (*c*)*.* The total number of medical and physical evaluations required to achieve statistical significance was 82 for the high-altitude group and 96 for the low-altitude control group. Through a non-probability convenience-based sampling technique, 117 participants were included in Limoncocha and 95 in Oyacachi.

### DNA extraction and analysis of ancestry ratios

To compare the ancestry of the two populations, a subsample of 47 unrelated individuals (30 Oyacachi vs 17 Limoncocha) were selected. We looked for ancestral differences among participants, and the results did not yield any significant difference [28].

### Data analysis

Descriptive statistics were used to analyze and visualize the differences between the two populations. Student’s *t*-test was used to analyze the differences between continuous variables, and the chi-square test was performed to check the association or independence of categorical variables. When the expected values were less than 5 in any of the categories, Fisher’s exact test or Spearman’s test were used when the variable had evident asymmetries with histograms prior to the selection of the test. The strength of the association between categorical variables was determined using the V-Cramer test.

All statistical analyses accepted significance for a *p*-value < 0.05. Calculations were completed using the IBM Corp. Released 2014; IBM SPSS Statistics for Windows, version 24.0. Armonk, NY: and R Core Team software 2018 version 3.5.1. Cartography was generated using QGIS Development Team 2.8, and all the references were managed using the open-source software Zotero 5.0.85.

## Results

A total of 212 subjects were recruited successfully in both communities. Of these, 55% (*n* = 117) were included from the Limoncocha low altitude group and 45% (*n* = 95) from the Oyacachi high altitude group. Women represented 63% (*n* = 134) from the entire cohort and men 37% (*n* = 78).

### Age and sex differences

Within this cohort, women from the low altitude group were on average 4 years older (41.0 [30.0–59.0]) than women from the high altitude group (36.0 [29.0–48.0]); nevertheless, this difference was not statistically significant (*p* = 0.121) (Table 1). Men in the low altitude cohort were on average 5 years older (42.0 [30.0–52.0]) than men living at high altitudes (36.0 [25.0–57.0]); similarity to women, this difference was not significant (*p* = 0.420).

**Table 1:** Sociodemographic, anthropometric, and risk factor analysis from the low and high cohorts

		Female				Male			
		Low altitude	High altitude	(%) Diff	Sig.	Low altitude	High altitude	(%) Diff	Sig.
Age (years), median (IQR)		41.0 (30.0–59.0)	36.0 (29.0–48.0)	13.0	0.121	42.0 (30.0–52.0)	36.0 (25.0–57.0)	15.4	0.420
Age categories	Young adult	45 (57.0)	41 (73.2)	9.3	0.086	24 (54.5)	27 (67.5)	11.7	0.475
	Adult	19 (24.1)	11 (19.6)	53.3	0.086	15 (34.1)	10 (25.0)	40.0	0.475
	Elderly	15 (19.0)	4 (7.1)	115.8	0.086	5 (11.4)	3 (7.5)	50.0	0.475
Weight (kg), mean ± SD		62.75 ± 14.44	60.84 ± 8.33	3.1	0.374	74.26 ± 10.83	60.34 ± 8.71	20.7	**0.000**
Height (cm), mean ± SD		149.22 ± 7.01	152.61 ± 8.62	2.3	0.333	159.90 ± 6.39	155.51 ± 9.93	2.8	**0.019**
BMI, mean ± SD		27.90 ± 5.10	26.10 ± 3.10	6.7	**0.022**	29.00 ± 4.20	24.90 ± 2.90	15.2	**0.000**
BMI categories	Underweight	0 (0.0)	0 (0.0)	0.0	**0.036**	0 (0.0)	0 (0.0)	0.0	**0.000**
	Normal	25 (32.1)	20 (35.7)	22.2	**0.036**	5 (12.5)	21 (53.8)	123.1	**0.001**
	Overweight	31 (39.7)	29 (51.8)	6.7	**0.036**	22 (55.0)	16 (41.0)	31.6	**0.002**
	Obesity	12 (15.4)	7 (12.5)	52.6	**0.036**	7 (17.5)	2 (5.1)	111.1	**0.003**
	Extreme obesity	10 (12.8)	0 (0.0)		**0.036**	6 (15.0)	0 (0.0)		**0.004**

### Weight (kg) and BMI

In relation to weight, we found that women at high altitudes (60.84 kg ± 8.333 kg) are on average 1.9 kg lighter than women at low altitudes (62.75 ± 14.44 kg), but this difference was not statistically significant (*p* = 0.374). Men living at high altitudes are 20.7% lighter than their counterparts at low altitudes (*p* = < 0.0001) (Table 1).

In terms of overweight, women living at high altitudes have a higher proportion (51.8%) of overweight subjects than those living at low altitudes (39.7%); however, for men, this relationship was reversed, with those living at low altitudes having a higher proportion (55%) of overweight subjects than those living at high altitude (41%).

For the measurement of obesity, the low altitude group in both men and women has a higher proportion of obese subjects (16.4%) than those subjects living at high altitudes (8.8%), being these differences statistically significant (Table 1). Concerning extreme obesity, we only found ten women and six men having extreme obesity (BMI > 40), belonging all from the low altitude setting (Table 1).

### Stature (cm)

**Fig. 2:**
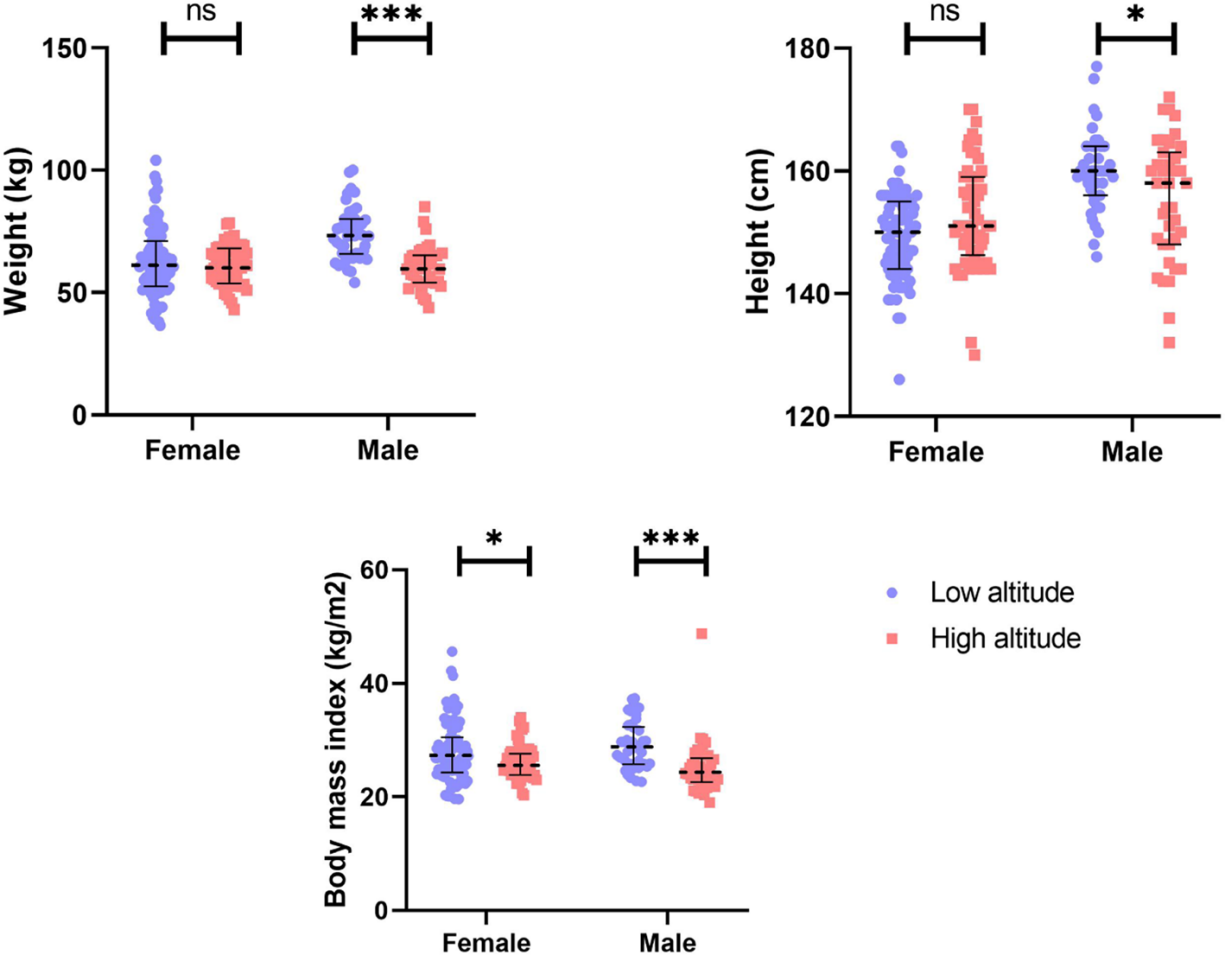
Weight, body mass index (BMI), and stature comparison among low and high altitude Kiwcha from Ecuador

In terms of stature, women from the high-altitude group are 3.3 cm taller (152.6 cm ± 8.62 cm) than women from the low altitude group (149.2 cm ± 7.01 cm); however, this difference was not statistically significant (*p* = 0.333). Among men, however, high altitude dwellers are 4.3 cm shorter (155.5 cm ± 9.93 cm) than lowlanders (159.9 cm ±6.39 cm), being this difference statistically significant (*p* = 0.019) (Fig. 2).

### Anthropometric characteristics

**Fig. 3:**
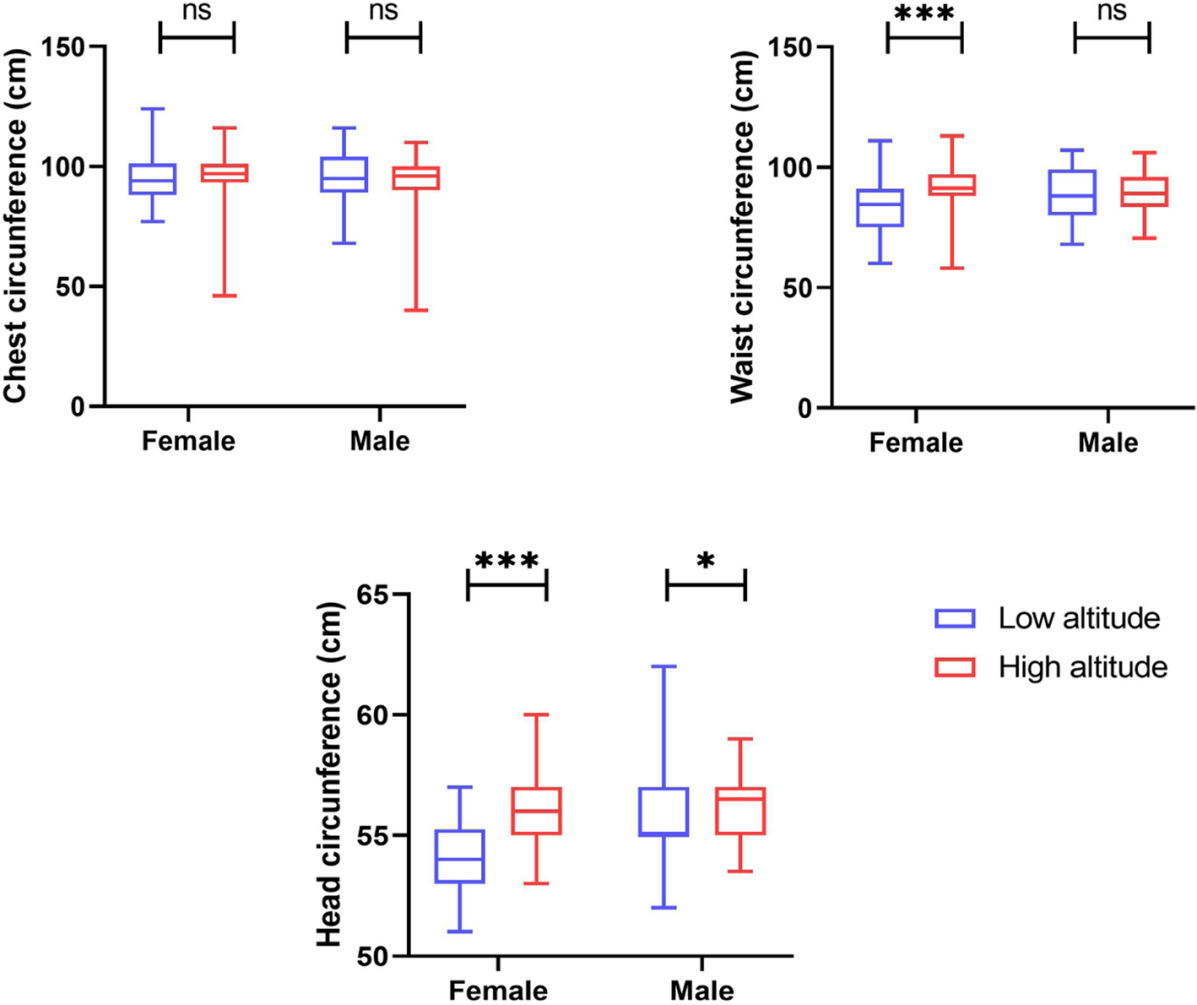
Chest, waist, and head circumference among low and high altitude men and women

Low altitude women are shorter (− 2.3%) and heavier (+ 3.1%) than women living at high altitude (Table 1). Shoulder height (− 0.3%), chest circumference (− 0.7%), and waist circumferences (− 9.1%) were also smaller in the low altitude group (Fig. 3).

### Head circumferences

We found that head circumference was significantly smaller among low altitude women (− 3.6%) and those women living at high altitude. Head circumference was also smaller for low altitude men (− 2.7%) (Table 2 and Fig. 3).

**Table 2:** Anthropometric measurements among low and high altitude dwellers

	Female				Male			
	Low altitude	High altitude	(%) Diff	Sig.	Low altitude	High altitude	(%) Diff	Sig.
Shoulder height, both arms (cm)	126.7 ± 6.8	127.1 ± 7.6	0.3	0.729	136.5 **±** 6.8	128.90**±** 8.2	5.7	**0.000**
Hip height (cm)	85.0 ± 4.5	84.1 ± 5.6	1.1	0.323	89.0 **±** 4.0	83.2 **±** 6.1	6.7	**0.000**
Buttock height (cm)	66.6 ± 3.7	65.4 ± 4.7	1.8	0.095	69.5 **±** 3.0	66.7 **±** 5.5	4.1	**0.011**
Lateral arm length (cm)	153.0 (148.0–156.0)	152.0 (149.0–160.0)	0.7	0.520	165.0 (158.0–175.0)	161.0 (151.0–167.0)	2.5	**0.048**
Shoulder height, one arm (cm)^a^	40.0 (37.0–41.0)	41.0 (39.0–45.0)	2.5	**0.002**	44.0 (43.0–46.0)	42.0 (40.0–44.0)	4.7	**0.004**
Bi-acromial shoulder width (cm)	52.0 (49.0–57.0)	50.0 (43.0–53.5)	3.9	**0.001**	52.0 (47.0–55.0)	49.0 (39.0–52.0)	5.9	**0.045**
Bi-iliac width (cm)	50.0 ± 8.0	49.00 ± 5.0	2.0	0.641	49.0 ± 5.0	48.0 ± 5.0	2.1	0.477
Arm length (cm)	66.1 ± 3.7	66.9 ± 6.5	1.2	0.417	70.8 ± 5.4	69.5 ± 5.1	1.9	0.268
Chest circumference (cm)	95.2 ± 10.1	95.9 ± 11.1	0.7	0.707	96.2 ± 10.1	94.2 ± 10.9	2.1	0.391
Waist circumference (cm)	84.1 ± 11.1	92.1 ± 8.70	9.1	**0.000**	89.4 ± 10.4	88.8 ± 9.1	0.7	0.782
Head circumference (cm)^a^	54.0 (53.0–55.0)	56.0 (55.0–57.0)	3.6	**0.000**	55.0 (55.0–57.0)	56.5 (55.0–57.0)	2.7	**0.012**
Body fat (%)	28.5 ± 6.6	33.3 ± 9.1	15.5	**0.002**	28.7 ± 6.7	28.7 ± 11.3	0.0	0.985
Body muscle (%)	36.3 ± 7.5	28.3 ± 6.7	24.8	**0.000**	29.0 ± 6.1	33.2 ± 8.3	13.5	**0.038**
Corporeal age (years)	29.0 ± 11.0	46.0 ± 14.0	45.3	**0.000**	36.0 ± 9.0	39.0 ± 17.0	8.0	0.523

^a^Median (IQR)

**Fig. 4:**
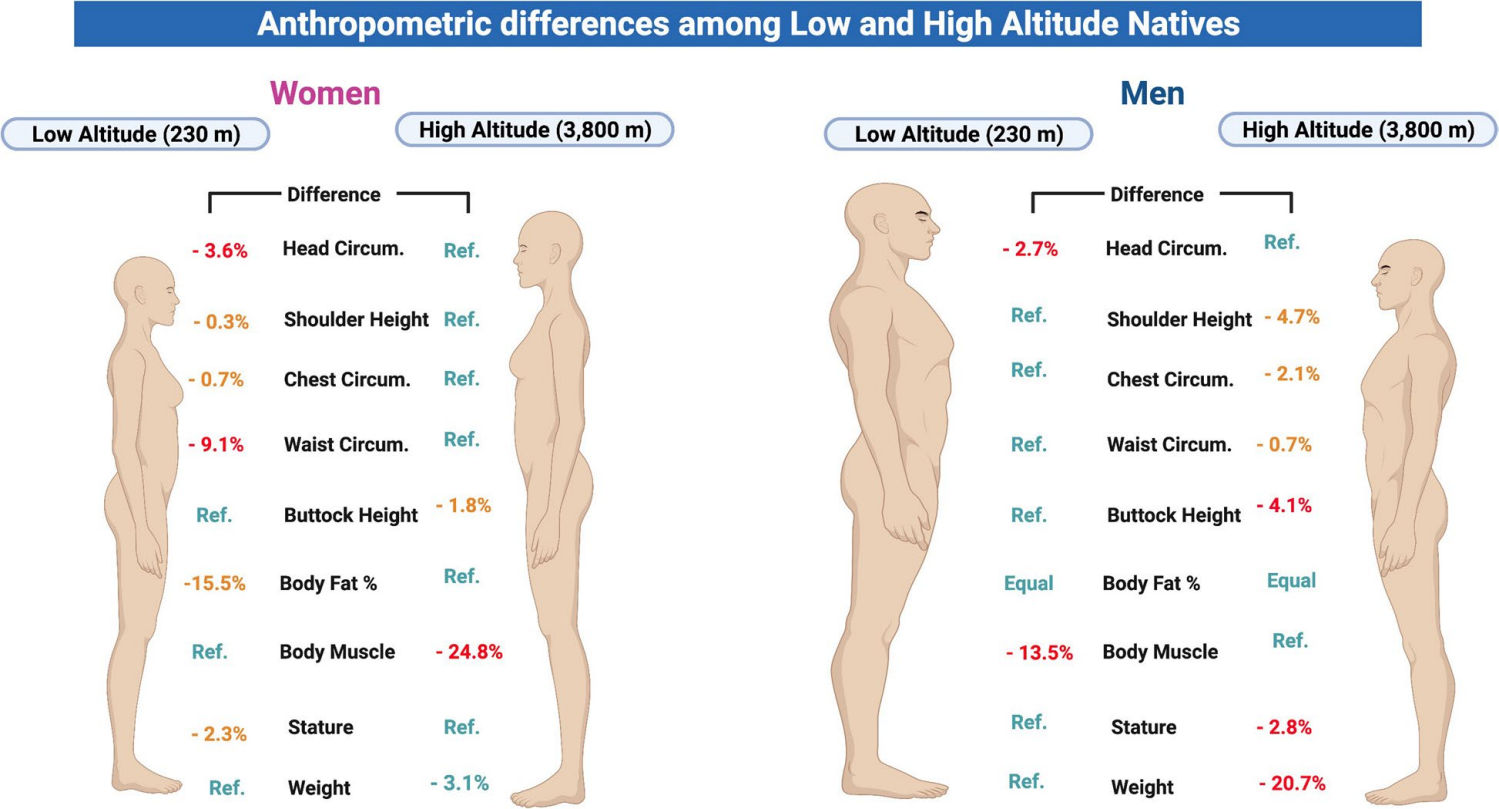
Anthropometric differences between Kiwcha men and women living at low and high altitudes

High altitude men have shorter shoulder height (− 4.7%), smaller chest circumference (− 2.1%) and waist circumference (− 0.7%), and shorter buttock height (− 4.1%) (Fig. 4).

### Body composition

**Fig. 5:**
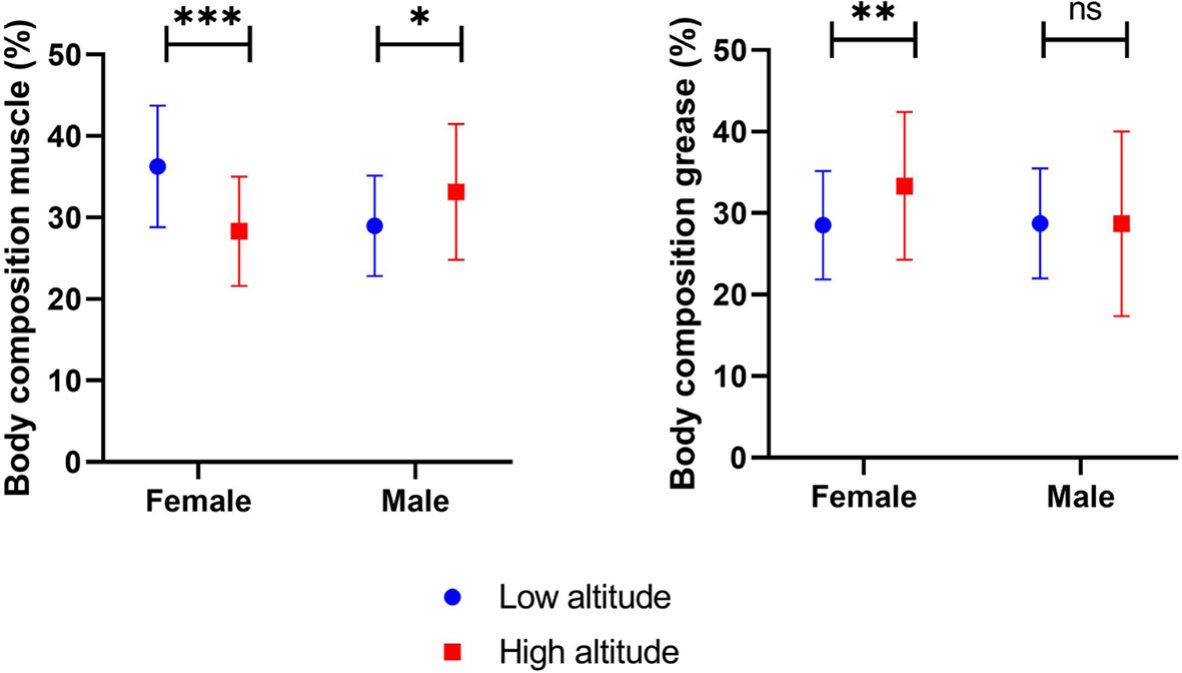
Muscle and fat body composition % among low and high altitude dwellers

In relation to body muscle percentage, women at high altitudes have less muscle (− 24.8%) mass than their counterparts at low altitudes, while men at high altitudes have significantly more muscle mass (+ 13.5%) than their lowland counterparts. Body fat percentage was lower among low altitude women (− 15.5%), and no differences were found among men (Fig. 5).

Body age was automatically calculated, and we found that high altitude women and men are 10 and 3 years older, respectively, than their actual age, while low altitude men and women are 3 and 12 years younger, respectively, than their actual age (Table 2).

### Age subgroup analysis

Comparing elderly participants of both communities, it was found that only one difference for each sex was present. Women in low altitude have smaller chest circumference (− 3.86%), and men have greater height (1.38%) compared to those higher altitudes. In the case of adults, low altitude women differ in bi-acromial shoulder width (+ 18.18%) and arm length (− 0.15%). But in the case of low altitude men, buttock height was higher (+ 5.71%) than those at high altitude. Finally, in the case of young adults, the most important findings were a higher BMI (+ 10.38%; + 15.42%) in low altitude women and men, respectively, compared to the high altitude groups (Supplementary Table S1).

## Discussion

The results of this study are the first to compare anthropometric differences in a genotype-controlled indigenous adult population living at low (230 m) and high altitude (3800 m) to our best knowledge. When analyzing the data, we observe that in general, women at high altitude are slightly lighter and slightly taller than women from the lowlands; nevertheless, high altitude men are significantly shorter and lighter than low altitude men (Fig. 4). The findings are similar to those reported in Bolivia by Leatherman et al. in 1984. This study conducted an anthropometric survey among 138 men from rural mountainous areas of Bolivia (3700 m) and concluded that high altitude men are shorter and lighter than their low altitude counterparts [29]. Among Quechuas, a similar native group from Peru, Toselli et al. in 2001 found shorter individuals at high altitudes in relationship to their corporeal mass [30]. In contrast to earlier findings, however, no evidence of these results was detected by Khalid in 1995 when he showed that high altitude residents from Saudi Arabia were significantly heavier and taller than the low altitude control group [31]. These differences between the two populations (the Andean and the Saudis) could demonstrate the differences in terms of adaptation, something that has been described extensively before [6, 19, 20, 32, 33].

It has been hypothesized that at least 5% of high altitude natives from Peru possess a newly discovered gene named *FBN1*. This gene seems to be associated with favoring high altitude Andean natives with low stature and possibly thicker skin [34]. Often high altitude dwellers and animals are smaller. An evolutionary response to the shortage of food or oxygen as well as thicker skin, which may help shield the body from intense UV radiation in such places [34, 35].

Weight among newborns is significantly lower among high-altitude neonates than the sea level counterparts has been shown previously [36, 37], a situation that might continue not only during pregnancy, but during the first years of childhood and adolescence [20, 21, 38].

The fact that newborns are smaller is due to an adaptive process that aims to reduce oxygen consumption by the fetus, being more efficient to deliver oxygen to a smaller organism throughout a smaller placenta [39–41].

Humans chronically exposed to high altitudes have compensated to the reduced partial pressure of oxygen (APO_2_) with anatomical and morphological/functional changes [42]. For instance, larger, wider, and deeper thoraxes and chests have been described among highlanders when they have been compared to low altitude dwellers [6, 43, 44]. This is probably due to the greater lung capacity of high altitude humans, especially those residing in the new world [45, 46]. Although this assertion has been demonstrated previously, in this study, it was found that there were no statistically significant differences in chest diameters, although women seem to have a slightly greater chest diameter in comparison with their lowlands counterparts.

In terms of anthropological differences, several authors have reported morphological findings that demonstrate adaptive differences among the inhabitants of the high altitudes. For instance, and besides chest diameters, weight, stature, and arm and leg lengths have been analyzed. Eichstaedt et al., in 2015, reported that arm length was shorter among high altitude natives, similar results to those found in our study [47].

In one report published on anthropometric differences among young natives, Pandey et al. 1990 reported that high altitude living is associated with a higher proportion of ectomorphic and mesomorphic than the low altitude group [48]. In these results, the group located at a higher altitude was prone to be overweight, especially among women, but in terms of obesity and extreme obesity, lowlanders reported a higher proportion of BMI > 30 (Table 1).

There are several reports showing that after acute exposure to high altitude, weight loss and loss of body fat percentage are evident [49]. In a study conducted by Zaccagni et al. in 2014, certain adaptive changes were evidenced after acute exposure to different altitudes (550 m to 5300 m). The authors reported that both sexes lost up to 4.0% of initial body mass, corresponding to 7.6% fat mass and 3.5% lean mass in men and 5.0% fat mass in women, as well as 3 to 6% lean mass in women [50]. They concluded that there is a significant acclimatization in terms of reduction of body mass measurements, regardless of the amount of physical activity performed. Despite these findings, in populations chronically residing at high altitude, the incidence of obesity appears to be lower with a significant increase in the percentage of muscle mass, as we also found it in our report. Long-term high-altitude exposure produces adaptive changes in numerous blood biochemical indicators, as well as a significant loss in body mass, including both lean and fat components [51]. This report shows there was a clear difference in trends between men and women in terms of body composition; whereas no difference was detected in body fat percentage in men, a significant higher fat accumulation is found in women at high altitude [52]. The presence of a low adiposity percentage among Quechua natives from Peru shows similar findings, especially for men. This lower body fat percentage could be associated with the stress of living at higher altitudes, as reported by Toselli et al. in 2001, findings that correlate to those previously reported by Bharadwaj et al. in 1981 [30, 53].

Very few studies in terms of bony structure differences have been conducted. Nevertheless, the very few that have measured head circumferences offer dissimilar results. In a study of Aymara children in Peru, it was found that the head circumference of high-altitude children was smaller than that of their low-altitude counterparts. But we found the opposite among high-altitude dwellers, having these significantly larger circumferences than the low altitude control [54].

Using bioelectrical impedance body meters, we have calculated a series of parameters that allowed us to calculate body composition (body fat percentage and body muscle percentage) as well as corporeal age [55–57]. These findings are noticeable; to our best knowledge, they are one of the very first reports to highlight this among high altitude populations. Significant differences between actual age and corporeal age among participants. We have found that low altitude dwellers, in general, have a body age that is on average 9 years younger than their actual average year; while body age among high altitude dwellers is significantly higher than their actual age in at least 6 years. These differences could be due to the hardness and the type of work performed at higher elevations and geographically remote areas. Steeper terrain, constant rainfall, and cold weather could have some association with these findings [58]. Also, this difference in aging could be one of the influence factors in age subgroup analysis findings, where body measures parameters were not stable between the groups. But also, it could be influenced by the limited sample of elder participants. On the other hand, another reason supporting the differences between men and women could be the role played by men versus women in both populations. For example, we have seen that women at high altitudes are generally heavier than those at low altitudes, but men are much more muscular and less overweight and obese than their counterparts at low altitudes. This could be explained by the arduous and laborious work that men do at high altitudes while women take care of children and domestic chores. For example, work at high altitudes is often related to agriculture, in some cases mining and in other cases tourism. The vast majority of these activities are carried out by men who have to carry heavy loads, which has been observed since 1950 [35]. Pugh’s observations on the Everest trek in 1952 and 1953 show that porters frequently carry weights of 40–50 kg, plus a 10-kg personal bag alone, for 10–12 h over 10–12 km per day. Ascents and descents of 1000–1200 m are common, with loads of tea or paper weighing more than 60 kg being carried on occasions [59]. At the Amazon basin, women must travel long distances to look for food and often contribute to activities related to fishing, gathering, and hunting [60, 61].

Another factor that has biological plausibility on influencing this “body aging,” seen among those living in Oyacachi at 3800 m, could be the effect of solar radiation that is greater at high altitudes, the chronic hypobaric hypoxia, and the possible effect that free radicals have on the muscles [62–64]. At the same time, it could be theorized that the great diversity of foods at low altitudes could contribute to improved absorption of antioxidants in the diet of those living at low altitudes. Although these assertions have little bibliographical support, they are findings that could lead to future research.

## Limitations

The main limitation of this study was the absence of a dietary and exercise assessment, as diet massively alters body composition and anthropometry. Also, despite obtaining a significant sample size to carry out this study, not all the populations belonging to these indigenous communities that met the inclusion criteria were willing to participate. So, even if it is a small probability, it cannot rule out that the inclusion of the data corresponding to those people who did not participate could produce variations in results or even alter interpretation. Another potential weakness is the gender asymmetry in the sample, with men totaling a fewer number of participants compared to numbers of women. Finally, the age of each subject was asked without cross-referencing with a valid governmental identification, thus, the veracity of the individual age cannot be absolutely guaranteed.

## Conclusion

The anthropometric differences vary according to sex, demonstrating that high altitude population is in general lighter and shorter than their low altitude controls. Men at high altitude, probably due to extenuating workloads, are lighter and have more muscled bodies than their lowland counterparts. Chest diameter and bi-acromial length were not greater among high-altitude dwellers as we expected. Finally, we found that body age is significantly higher than their real age among high-altitude populations, while low altitude populations have younger body age than their actual age, possibly linked to the climatic and sociodemographic conditions found in these locations. Further study on this subject is needed to strengthen the evidence.

## Supplementary Information

**Additional file 1.** Supplementary table.
