# Supplementary material for: Genotyped indigenous Kiwcha adults at high altitude are lighter and shorter than their low altitude counterparts
Source: J Physiol Anthropol. 2022 Mar 10;41:8. doi: 10.1186/s40101-022-00280-6 (PMC8908589; doi:10.1186/s40101-022-00280-6)
Supplement: Supplementary file 1 — Additional file 1. Supplementary table. [file 40101_2022_280_MOESM1_ESM.docx]

| Sex | Female | | | | | | | | | Male | | | | | | | | |
| --- | --- | --- | --- | --- | --- | --- | --- | --- | --- | --- | --- | --- | --- | --- | --- | --- | --- | --- |
| Age categories | Young Adult | | | Adult | | | Elderly | | | Young Adult | | | Adult | | | Elderly | | |
| Characteristics / Altitude | Low Altitude (252 m) | High Altitude (3,800 m) | p value | Low Altitude (252 m) | High Altitude (3,800 m) | p value | Low Altitude (252 m) | High Altitude (3,800 m) | p value | Low Altitude (252 m) | High Altitude (3,800 m) | p value | Low Altitude (252 m) | High Altitude (3,800 m) | p value | Low Altitude (252 m) | High Altitude (3,800 m) | p value |
| Weight (Kg) | 66,99 (± 13,16) | 60,44 (± 8,41) | 0.510 | 64,14 (± 14,65) | 62,08 (± 9,43) | 0.168 | 48,27 (± 7,54) | 61,45 (± 4,87) | 0.229 | 72,37 (± 10,50) | 58,16 (± 6,93) | 0.149 | 76,51 (± 12,31) | 63,35 (± 9,15) | 0.505 | 75,84 (± 7,19) | 69,23 (± 15,46) | 0.259 |
| Height (cm) | 152,56 (± 5,14) | 152,48 (± 8,57) | **0.031** | 147,16 (± 6,31) | 153,05 (± 10,56) | 0.155 | 141,80 (± 6,25) | 152,75 (± 3,30) | 0.472 | 161,41 (± 5,10) | 156,21 (± 9,42) | **0.000** | 160,87 (± 6,31) | 155,35 (± 10,20) | 0.076 | 150,40 (± 3,65) | 150,00 (± 15,62) | **0.010** |
| Shoulder height, both arms | 129,5 (± 5,4) | 127,0 (± 7,9) | **0.000** | 124,7 (± 5,7) | 128,4 (± 7,6) | 0.512 | 120,5 (± 7,0) | 124,5 (± 4,9) | 0.555 | 137,1 (± 5,8) | 129,4 (± 7,7) | 0.058 | 137,9 (± 7,3) | 128,4 (± 8,6) | 0.465 | 130,2 (± 6,8) | 126,0 (± 13,9) | 0.117 |
| Hip height | 86,6 (± 4,0) | 83,8 (± 5,5) | **0.008** | 84,3 (± 3,9) | 85,5 (± 5,1) | 0.113 | 81,2 (± 4,5) | 84,5 (± 8,5) | 0.128 | 89,5 (± 4,0) | 83,2 (± 5,6) | 0.057 | 89,9 (± 4,6) | 82,6 (± 7,1) | 0.168 | 84,4 (± 5,2) | 85,3 (± 8,1) | 0.298 |
| Buttock height | 67,2 (± 3,7) | 65,2 (± 4,2) | 0.083 | 66,1 (± 3,5) | 66,2 (± 6,6) | 0.121 | 65,5 (± 4,0) | 64,8 (± 4,6) | 0.619 | 69,4 (± 3,5) | 66,4 (± 4,7) | 0.171 | 70,9 (± 3,2) | 67,2 (± 6,1) | **0.044** | 65,6 (± 5,2) | 67,7 (± 10,7) | 0.190 |
| Lateral arm length | 154,9 (± 7,7) | 154,3 (± 10,9) | 0.518 | 152,4 (± 8,9) | 153,9 (± 8,8) | 0.841 | 147,1 (± 7,2) | 150,8 (± 7,8) | 0.946 | 167,0 (± 8,4) | 160,7 (± 11,7) | 0.114 | 165,9 (± 10,8) | 158,5 (± 9,7) | 0.936 | 152,8 (± 11,0) | 156,7 (± 11,4) | 0.896 |
| Shoulder Height, one arm | 40 (± 3) | 42 (± 4) | 0.146 | 40 (± 3) | 42 (± 4) | 0.153 | 37 (± 2) | 40 (± 2) | 0.362 | 44 (± 2) | 42 (± 4) | 0.066 | 45 (± 3) | 44 (± 4) | 0.454 | 42 (± 3) | 41 (± 3) | 0.678 |
| Bi-acromial Shoulder Width | 53,3 (± 5,9) | 48,1 (± 8,0) | **0.047** | 53,3 (± 4,2) | 45,1 (± 9,9) | **0.002** | 50,0 (± 5,8) | 50,8 (± 12,7) | 0.067 | 49,0 (± 3,3) | 47,1 (± 8,5) | **0.000** | 53,6 (± 4,1) | 49,7 (± 7,3) | 0.280 | 51,4 (± 6,1) | 38,3 (± 7,6) | 0.524 |
| Bi-iliac width | 49 (± 6) | 49 (± 5) | 0.130 | 53 (± 10) | 47 (± 5) | 0.340 | 49 (± 7) | 52 (± 4) | 0.294 | 46 (± 3) | 47 (± 5) | 0.328 | 51 (± 5) | 49 (± 4) | 0.849 | 52 (± 6) | 48 (± 1) | 0.141 |
| Arm length | 67,2 (± 3,2) | 67,4 (± 6,6) | 0.367 | 65,8 (± 3,2) | 65,9 (± 7,1) | **0.001** | 63,4 (± 4,4) | 64,5 (± 4,1) | 0.756 | 72,0 (± 3,3) | 69,8 (± 5,3) | 0.104 | 71,5 (± 4,0) | 69,9 (± 4,8) | 0.458 | 64,4 (± 10,4) | 65,3 (± 4,0) | 0.294 |
| Chest circumference | 96,4 (± 10,1) | 96,6 (± 9,6) | **0.000** | 97,6 (± 10,1) | 96,9 (± 7,2) | 0.241 | 88,9 (± 7,7) | 85,6 (± 26,5) | **0.002** | 92,9 (± 8,1) | 92,7 (± 11,9) | 0.944 | 99,3 (± 8,4) | 99,0 (± 6,4) | 0.275 | 99,2 (± 18,3) | 90,7 (± 10,7) | 0.577 |
| Waist circumference | 83,5 (± 11,8) | 91,1 (± 8,9) | 0.149 | 87,3 (± 10,3) | 95,8 (± 8,2) | 0.417 | 82,3 (± 9,7) | 92,3 (± 4,7) | 0.077 | 84,4 (± 9,4) | 87,0 (± 9,7) | 0.647 | 93,1 (± 9,3) | 92,5 (± 7,3) | 0.456 | 97,2 (± 9,5) | 91,5 (± 6,4) | 0.535 |
| Head circumference | 54,7 (± 1,4) | 56,0 (± 1,5) | **0.013** | 54,2 (± 1,7) | 56,2 (± 1,6) | 0.815 | 53,6 (± 1,2) | 55,8 (± 1,3) | 0.822 | 55,2 (± 1,3) | 56,3 (± 1,3) | 0.815 | 55,7 (± 1,7) | 56,6 (± 1,8) | 0.870 | 56,6 (± 3,2) | 56,5 (± ,9) | 0.195 |
| BMI | 28,7 (± 5,3) | 26,0 (± 3,0) | 0.927 | 29,0 (± 4,8) | 26,5 (± 3,5) | 0.254 | 23,9 (± 2,8) | 26,5 (± 2,9) | 0.931 | 27,7 (± 4,2) | 24,0 (± 2,6) | **0.013** | 29,4 (± 3,4) | 27,2 (± 2,2) | 0.390 | 33,5 (± 2,9) | 24,9 (± 3,3) | 0.750 |
| Body composition grease% | 28,8 (± 7,3) | 33,6 (± 9,1) | **0.002** | 29,5 (± 5,1) | 32,1 (± 7,5) | 0.161 | 26,0 (± 6,4) | 33,5 (± 14,1) | 0.135 | 27,9 (± 4,8) | 27,1 (± 11,1) | **0.000** | 30,5 (± 8,7) | 31,6 (± 11,8) | 0.104 | 24,7 (± 6,4) | 32,6 (± 13,3) | 0.183 |
| Body composition Muscle% | 35,8 (± 7,3) | 28,2 (± 7,0) | 0.068 | 38,9 (± 7,8) | 28,0 (± 4,6) | 0.138 | 33,8 (± 7,0) | 30,4 (± 10,1) | 0.496 | 30,6 (± 6,8) | 33,9 (± 8,6) | **0.014** | 26,9 (± 5,2) | 32,1 (± 7,8) | 0.201 | 28,8 (± 0) | 29,8 (± 9,2) | **0.019** |
| Corporal Age | 30 (± 13) | 45 (± 14) | 0.681 | 27 (± 7) | 49 (± 17) | **0.010** | 30 (± 14) | 44 (± 17) | 0.496 | 35 (± 8) | 37 (± 17) | **0.003** | 39 (± 11) | 42 (± 16) | 0.457 | 30 (± 0) | 47 (± 30) | **0.000** |
| Real Age | 31 (± 8) | 41 (± 15) | 0.144 | 51 (± 11) | 52 (± 19) | **0.019** | 69 (± 14) | 38 (± 7) | 0.525 | 29 (± 9) | 34 (± 14) | 0.215 | 52 (± 6) | 37 (± 15) | **0.005** | 77 (± 5) | 46 (± 27) | **0.000** |
